# Additional mesenchymal stem cell injection improves the outcomes of marrow stimulation combined with supramalleolar osteotomy in varus ankle osteoarthritis: short-term clinical results with second-look arthroscopic evaluation

**DOI:** 10.1186/s40634-016-0048-2

**Published:** 2016-05-20

**Authors:** Yong Sang Kim, Moses Lee, Yong Gon Koh

**Affiliations:** Department of Orthopaedic Surgery, Center for Stem Cell & Arthritis Research, Yonsei Sarang Hospital, 478-3, Bangbae-dong, Seocho-gu Seoul, Korea

**Keywords:** Mesenchymal stem cell, Marrow stimulation, Supramalleolar osteotomy, Varus ankle osteoarthritis

## Abstract

**Background:**

Supramalleolar osteotomy (SMO) is reported to be an effective treatment for varus ankle osteoarthritis by redistributing the load line within the ankle joint. Mesenchymal stem cells (MSCs) have been proposed as a new treatment option for osteoarthritis on the basis of their cartilage regeneration ability. The purpose of this study was to compare the clinical, radiological, and second-look arthroscopic outcomes between MSC injection with marrow stimulation and marrow stimulation alone in patients with varus ankle osteoarthritis who have undergone SMO.

**Methods:**

In this retrospective study, 62 patients (64 ankles) with varus ankle osteoarthritis underwent second-look arthroscopy at a mean of 12.8 months after arthroscopic marrow stimulation combined with SMO; 33 ankles were subjected to marrow stimulation alone (group I), and 31 were subjected to marrow stimulation with MSC injection (group II). Clinical outcome measures included a visual analog scale (VAS) for pain and the American Orthopaedic Foot and Ankle Society (AOFAS) score. Radiological outcome variables included the tibial–ankle surface (TAS), talar tilt (TT), and tibial–lateral surface (TLS) angles. In second-look arthroscopy, cartilage regeneration was evaluated using the International Cartilage Repair Society (ICRS) grade.

**Results:**

The mean VAS score improved significantly from 7.2 ± 1.0 to 4.7 ± 1.4 in group I and from 7.3 ± 0.8 to 3.7 ± 1.5 in group II at the final follow-up (*P* < 0.001 for both groups). The mean AOFAS score also improved significantly from 61.7 ± 5.8 to 80.9 ± 6.7 in group I and from 60.6 ± 6.1 to 85.2 ± 5.1 in group II at the final follow-up (*P* < 0.001 for both groups). There were significant differences in the mean VAS and AOFAS scores between groups at the final follow-up (*P* = 0.002 and 0.010, respectively). At second-look arthroscopy, there were significant differences in ICRS grades between groups(*P* = 0.015 for medial aspect of the talar dome, *P* = 0.044 for medial aspect of the tibial plafond, and *P* = 0.005 for articular surface of the medial malleolus). ICRS grades were significantly correlated with clinical outcomes in both groups (all *P* < 0.05). Mean TAS, TT, and TLS angles improved significantly after SMO in both groups but were not significantly correlated with clinical outcomes or ICRS grade (all n.s.).

**Conclusions:**

The clinical and second-look arthroscopic outcomes of MSC injection with marrow stimulation were better compared to those of marrow stimulation alone in patients with varus ankle osteoarthritis who have undergone SMO. Furthermore, the ICRS grade is significantly correlated with clinical outcome.

## Background

Supramalleolar osteotomy (SMO) has been reported to successfully restore normal joint function or halt further disease progression in varus ankle osteoarthritis (Cheng et al. 2001; Kim et al. 2014b; Pagenstert et al. 2007; Tanaka et al. 2006). SMO aims to alter the joint mechanics in a varus ankle by correcting the medial displacement of the load line and shifting the medial concentration of the stress laterally onto the intact articular cartilage of the lateral side within the ankle (Becker and Myerson 2009; Castagnini et al. 2016; Cheng et al. 2001). However, although SMO can alter the weight-bearing axis, which provides an ideal mechanical environment for halting degenerative changes in the articular cartilage, the overall long-term success of SMO remains debatable if cartilage regeneration of medial osteoarthritic lesions is not achieved.

Although cartilage regenerative procedures such as marrow stimulation are not traditional treatment measures for osteoarthritis, they are gaining increasing interest because of their potential to provide pain relief and alter osteoarthritis progression (Lyu et al. 2012; Zhu et al. 2013). Therefore, a marrow stimulation procedure for arthritic lesions in the medial aspect of the ankle joint may improve the overall outcomes of SMO in varus ankle osteoarthritis. Kim et al (Kim et al. 2014b) performed arthroscopic marrow stimulation along with SMO in patients with varus ankle osteoarthritis and assessed cartilage regeneration after arthroscopic marrow stimulation using second-look arthroscopic evaluation; the cartilage regeneration of medial osteoarthritic lesions was significantly associated with the clinical outcomes of SMO. Therefore, they concluded that arthroscopic marrow stimulation should be considered with SMO to ensure adequate cartilage regeneration of the medial aspect of the ankle joint. Marrow stimulation treatment primarily aims to recruit mesenchymal stem cells (MSCs) from bone marrow, which leads to coverage of the lesion with fibrous cartilage (Giannini et al. 2009; Hangody et al. 2001; Kono et al. 2006). This treatment provides acceptable clinical results over midterm follow-up periods but often fail in the long term because of biomechanical insufficiency of the regenerative fibrous cartilage and scar tissue that results from this method (Baltzer and Arnold 2005). Recently, MSCs have been suggested for use in the cell based treatment of cartilage lesions. Regarding in vitro studies, the application of MSCs into full-thickness articular cartilage defects has been attempted under various conditions, and MSCs have been used with success in hybrid scaffolds to repair cartilage lesions in animal models (Han et al. 2008; Kobayashi et al. 2008). Moreover, MSCs were recently proposed as a new treatment option for osteoarthritis on the basis of their ability to differentiate into chondrocytes as well as the paracrine effects of their secreted bioactive materials (Barry and Murphy 2013; Beris et al. 2005; Caplan and Dennis 2006; Galois et al. 2005; Oreffo et al. 2005).

We hypothesized that the additional injection of MSCs would improve the outcomes of marrow stimulation in patients with varus ankle osteoarthritis who have undergone SMO, because of their capability to differentiate into articular cartilage as a result of injection into cartilage lesions. The purpose of this study was to compare the clinical, radiological, and second-look arthroscopic outcomes between MSC injection with marrow stimulation and marrow stimulation alone in patients with varus ankle osteoarthritis who have undergone SMO.

## Methods

### Patient enrollment

**Fig. 1:**
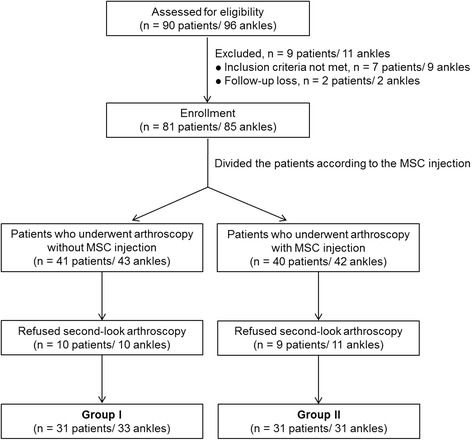
Flow diagram of patient involvement in the study

This retrospective comparative study was designed to evaluate the effectiveness of MSC injection in patients who underwent marrow stimulation with SMO for varus ankle osteoarthritis. The study protocol was approved by our institutional review board, andwritten informed consentwas obtained from all study participants. In this study, the indication for SMO was stage 2 or 3A osteoarthritis according to the Takakura classification system as modified by Tanaka et al. (Tanaka et al. 2006). Patients with a history of surgical treatments or arthritic changes in the entire ankle joint, deformity proximal to the ankle as seen on plain radiographs, or varus deformity of the hind foot as seen in the heel alignment view were excluded. From May 2009 to September 2013, 90 consecutive patients including 96 ankles with medial ankle osteoarthritis and varus deformity underwent SMO with arthroscopic marrow stimulation. Among 90 patients, 9 patients were excluded and 81 patients were enrolled in this study. Patients were informed about MSCs preoperatively and decided whether to receive them at the time of surgery; the decision was solely up to the patients. Moreover, before surgery, we recommended second-look arthroscopy to all patients and explained that its purpose was to evaluate the medial arthritic lesion and that this would require additional arthroscopic procedures such as debridement, synovectomy, and adhesiolysis. Among the 81 patients (85 ankles), second-look arthroscopy was performed at a mean of 12.8 months postoperatively (range, 9 to 16 months) in 64 of the 85 ankles; these 64 ankles in 62 patients were ultimately included in the present study. Among these 64 ankles, 33 underwent arthroscopic marrow stimulation alone (group I), and 31 underwent arthroscopic marrow stimulation with MSCs injection (group II) (Fig. 1).

### Tissue collection and isolation of cells

Subcutaneous adipose tissue was harvested from both buttocks of each patient. One day before arthroscopic surgery, adipose tissue was harvested by tumescent liposuction while the patient under local anesthesia (Klein 1996). We aimed to routinely collect 140 cc liposuctioned adipose tissue: 120 cc was used for the injection, and the remaining 20 cc was analyzed to examine the plastic-adherent cells that form fibroblast colony-forming units (CFU-F) and confirm the multilineage differentiation of adipose-derived stem cells.

In the operating room, 120 cc adipose tissue was suspended in phosphate-buffered saline, placed in a sterile box, and transported to the laboratory. Mature adipocytes and connective tissues were separated from the stromal vascular fraction by centrifugation as described by Zuk et al. (Zuk et al. 2001). The remaining 20 cc adipose tissue was processed in the same manner and used for cell analysis.

### Epitope profile and differentiation assay

To evaluate the frequency of mesenchymal-like progenitors in the stromal vascular fraction, cells were cultured in T-25 flasks at a final concentration of 16 cells/cm^2^. Colonies consisting of ≥50-cell aggregates were scored under an optical microscope, and the immunophenotype of the adipose-derived stem cells was analyzed by flow cytometry (i.e., FACS) analysis. MSC marker phenotyping was performed using CD14, CD34, CD90, and CD105 antibodies as described previously (Marchal et al. 2012). CD14 and CD34 are hematopoietic cell markers, and CD90 and CD105 are mesenchymal cell markers. Adipose-derived stem cells were plated at cells/cm^2^ in Dulbecco’s modified Eagle medium containing 10 % fetal bovine serum and were allowed to adhere for 24 h. The culture medium was subsequently replaced with specific inductive media to determine the adipogenic, osteogenic, and chondrogenic differentiation potential as described previously (Marchal et al. 2012).

### Surgical procedures and MSC application

**Fig. 2:**
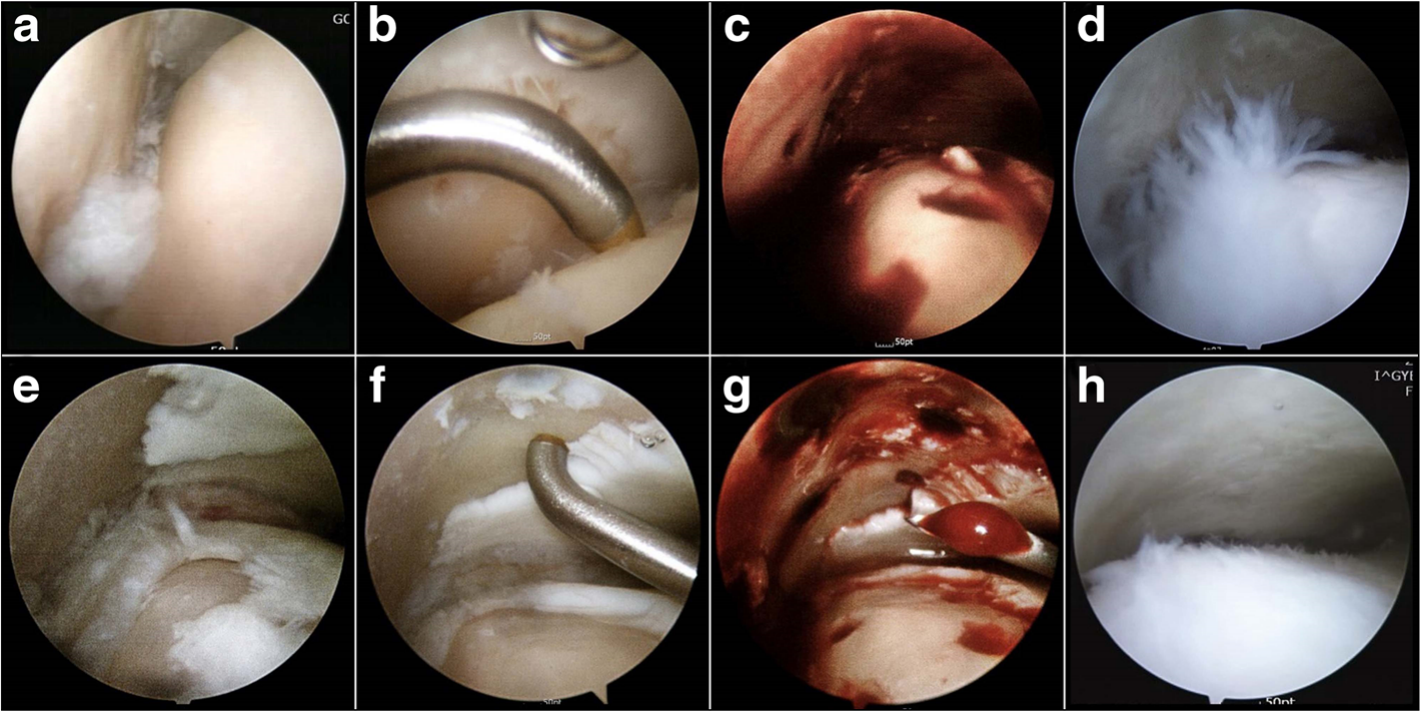
**a**–**d** Arthroscopic views of left ankle in a 54-year-old male patient who underwent marrow stimulation alone combined with supramalleolar osteotomy. **e**–**h** Arthroscopic views of the left ankle in a 52-year-old female patient who underwent mesenchymal stem cell injection and marrow stimulation combined with supramalleolar osteotomy. **a**, **e** Intraoperative arthroscopic findings showing a cartilage lesion with osteoarthritic change in the medial gutter area. **b**, **f** Arthroscopic views showing the perforation of the subchondral bone with a microfracture awl after the debridement of all unstable and damaged cartilage in the lesion. **c**, **g** Adequate bone bleeding at microfracture holes was confirmed after the tourniquet was released. **d** Second-look arthroscopy showing poor cartilage regeneration at the microfractured site (International Cartilage Repair Society [ICRS] grade IV). **g** Arthroscopic view after mesenchymal stem cell injection. **h** Second-look arthroscopy showing nearly normal coverage of the cartilage lesion (ICRS grade II)

The patient was placed in the supine position under spinal anesthesia. A thigh tourniquet was used for hemostasis. Non-invasive ankle distraction (15 pounds) was applied using an ankle harness. An anteromedial portal adjacent to the anterior tibial tendon and an anterolateral portal adjacent to the peroneus tertius tendon were used. A 2.7-mm arthroscope with a 30° angle was used for arthroscopic evaluation. In all cases, the arthritic lesions of the articular surface of the medial malleolus, medial aspect of the tibial plafond and/or medial aspect of the talar dome were noted during the arthroscopic procedures. The arthroscopic marrow stimulation procedure for these arthritic lesions was performed in a standardized manner by a single surgeon (Y.S.K.). After thorough debridement of all unstable and damaged cartilage in the lesion, a microfracture or abrasion arthroplasty was performed. For the microfracture, multiple perforations perpendicular to the joint surface were made using a 2.5-mm 90° microfracture awl (Linvatec, Largo, FL, USA) as described by Steadman et al. (Steadman et al. 2001). For areas showing a loss of subchondral bone, abrasion arthroplasty was performed by removing loose or osteochondral fragments with a ring-shaped or curved curette and by trimming damaged cartilage with a power shaver until a stable and smooth articular surface was achieved. The tourniquet was subsequently released, and adequate bone bleeding at the microfracture holes or abrasion arthroplasty site was confirmed. For group 2, MSCs isolated 1 day preoperatively were injected after the extraction of arthroscopic fluid within the joint (Fig. 2).

After the arthroscopic procedure, SMO was performed as follows. After the level of the osteotomy was determined by using an image intensifier, a skin incision was made on the medial side of the distal tibia centered over the osteotomy site. Periosteal stripping was performed, and a Kirschner wire was placed proximal to the tip of the medial malleolus to identify the osteotomy plane; it was inserted obliquely toward the proximal margin of the syndesmosis. The osteotomy was made using a broad oscillating saw to preserve the opposite cortex, acting as a fulcrum for the opening wedge and enhancing stability. The deformity was carefully corrected by the stepwise insertion of 2 or 3 osteotomes to avoid far cortex fractures. Alignment was assessed using an image intensifier, and the appropriate size of the opening wedge was determined on the basis of the preoperatively planned correction. Although, the normal values of the tibial–ankle surface (TAS) angle and the correction angle are debated, (Knupp et al. 2011; Lee et al. 2011; Tanaka 2012) Sugimoto et al. (Sugimoto et al. 1997) report that the mean TAS and tibial–lateral surface (TLS) angles in healthy Japanese people are 88° and 81°, respectively; therefore, we attempted to achieve these angles in the present study. The osteotomy was subsequently stabilized using 2 single-opening wedge plates and screws (B. Braun Aesculap, Tuttlingen, Germany), and the osteotomy gap was filled with cancellous bone allograft. Postoperatively, a short leg splint was applied for 2 weeks. Following suture removal, a non-weight-bearing short leg cast was applied for 4 weeks. Active and passive ankle range of motion exercises were initiated 6 weeks postoperatively. Sports and high-impact activities were limited for at least 3 months postoperatively.

### Outcome assessments

All patients were clinically evaluated preoperatively and during follow-up. A visual analog scale (VAS) pain score and the American Orthopaedic Foot and Ankle Society (AOFAS) ankle-hind foot score were used for clinical evaluations.

**Fig. 3:**
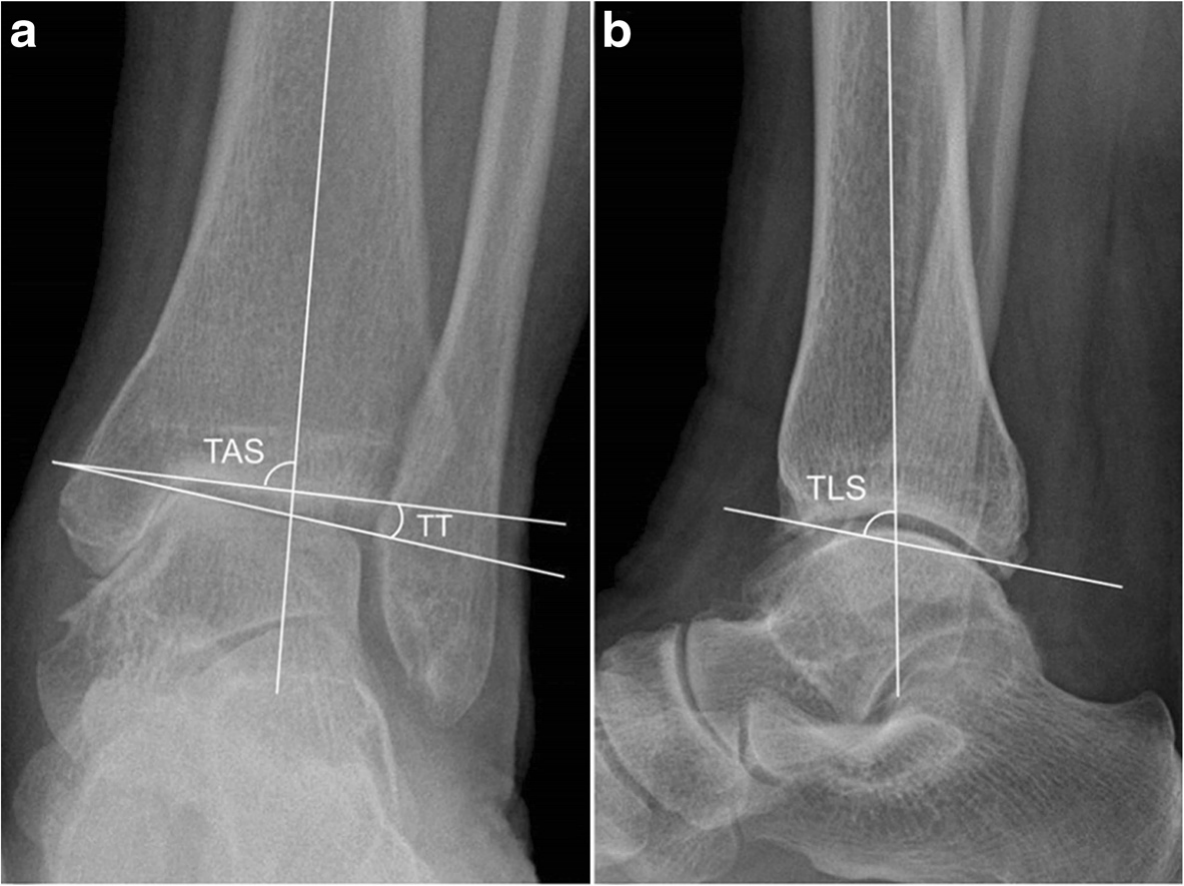
Weightbearing ankle radiographs showing the radiographic parameters used to measure alignment in ankles with varus ankle osteoarthritis. **a** Anteroposterior view showing measurement of the tibial-ankle surface angle (TAS) and the talar tilt (TT). **b** Lateral view showing measurement of the tibial-lateral surface angle (TLS)

Weight-bearing anteroposterior and lateral radiographs were obtained for radiological evaluation preoperatively and during follow-up. On the weight-bearing anteroposterior radiograph, the TAS angle was defined as the angle between the tibial axis and the tibial plafond, and the talar tilt (TT) angle was defined as the angle between the tibial plafond and the talar dome (Fig. 3) (Kim et al. 2014b). On the weight-bearing lateral radiograph, the TLS angle was defined as the angle between the axis of the tibia and a line drawn between the anterior and posterior margins of the tibial plafond to mark the articular surface of the distal aspect of the tibia (Fig. 3) (Kim et al. 2014b). To avoid potential bias, an independent observer, who was a trained musculoskeletal radiologist uninvolved in patient care and unaware of the study objectives, evaluated the radiographs. At serial follow-up examinations, the osteotomy sites were examined by plain radiography.

### Second-look arthroscopic evaluation

The plates and screws were removed after radiological and clinical confirmation of the union of the osteotomy site. All second-look arthroscopies were performed by a single surgeon (Y.S.K.) when the plates and screws that fixed the open wedge osteotomy sites were removed.

Cartilage lesions were macroscopically evaluated after arthroscopic marrow simulation according to the International Cartilage Repair Society (ICRS) (Brittberg and Peterson 1998; Peterson et al. 2000) grade. To evaluate the cartilage lesions according to location, we divided the cartilage lesions into 3 areas on the medial aspect of the ankle joint: arthritic lesions of the medial aspect of the talar dome, medial aspect of tibial plafond, and articular surface of the medial malleolus. The ICRS grading system involves 3 criteria: the degree to which a defect is filled by repair tissue, degree of integration of the repair tissue with the adjacent articular cartilage, and macroscopic appearance of the surface of the repair site. These arthroscopic evaluation criteria were subjectively scored from 0 to 4; they were then combined for an overall grade from I to IV, which correspond to normal, nearly normal, abnormal, and severely abnormal, respectively (Table 1). The ICRS grading system is reported to be a reliable and relevant means of macroscopically evaluating cartilage regeneration after microfracture or autologous chondrocyte implantation (van den Borne et al. 2007). Additional arthroscopic procedures such as synovectomy, adhesiolysis, and debridement of the impinged soft tissue were performed if pathologic lesions were found in the ankle joint during the second-look arthroscopy.

**Table 1:** Comparison of the baseline demographics in both groups

	Group I	Group II	*P* Value
Ankles/patients, n	33/31	31/31	
Age, y	51.4 ± 8.5	52.2 ± 5.9	n.s.
Sex, male/female, n	14/17	15/16	n.s.
Body mass index, kg/m^2^	26.8 ± 3.3	27.1 ± 3.2	n.s.
Side of involvement, right/left, n	15/18	17/14	n.s.
Follow-up, mo	26.6 ± 4.0	27.6 ± 5.0	n.s.

Values are expressed as mean ± standard deviation unless otherwise indicated

### Statistical analysis

The sample size was 64 and descriptive statistics were presented as mean ± standard deviation (SD). The principal dependent variables were VAS and AOFAS scores at the final follow-up as clinical outcomes; postoperative TAS, TT, and TLS angles as radiological outcomes; and ICRS grade at second-look arthroscopy. For the evaluation of the magnitudes of the differences in the outcome scores and whether these were likely to be clinically significant, we used a distribution based method to calculate the minimal clinically importance difference (MCID). According to the distribution-based method measuring the variability of the standard error of measurement (SEM), we compared the change in clinical outcomes (VAS and AOFAS score). The SEM is the variation in scores due to unreliability of the scale or measure used. Therefore, a change smaller than the SEM is likely to be the result of measurement error rather than a true observed change. Patients achieving a difference in outcome score of at least one SEM would have achieved a MCID (Copay et al. 2007). The Wilcoxon signed-rank test was used to evaluate differences between preoperative and final follow-up values, while the Mann–Whitney *U*-test was used to compare results between groups. The *χ*^2^ test or Fisher’s exact test was used to compare categorical data where appropriate. The Spearman rank-order correlation test was used to determine the correlations between ICRS grades at second-look arthroscopy and clinical outcomes at the final follow-up. The correlations of postoperative radiological outcomes with clinical outcomes at the final follow-up and ICRS grades were also analyzed using the Spearman rank-order correlation test. A *P* value of < 0.05 was considered statistically significant.

## Results

### General characteristics

Our final sample size was 62 patients (64 ankles; 31 men, 31 women). The general characteristics of the study population are summarized in Table 1. There were no significant differences between groups with respect to age, sex, body mass index, or follow-up period.

### Isolation and characterization of MSCs

We evaluated the capacity of human subcutaneous adipose tissue to generate mesenchymal progenitors according to CFU-F. Thus, after isolation, the adipose-derived stem cells represented a mean of 8.7 % of the stromal vascular fraction cells (range, 6.9 to 11.2 %). After the stromal vascular fractions were isolated, a mean of 4.0 × 10^6^ stem cells (8.7 % of 4.6 × 10^7^ stromal vascular fraction cells; range, 3.2 to 5.2 × 10^6^ cells) were prepared. Accordingly, a mean of 4.8 × 10^7^ stromal vascular fraction cells, which contained a mean of 4.0 × 10^6^ stem cells, were used for MSC injection. FACS characterization indicated positive expressions of the surface markers CD90 (98.42 %) and CD105 (92.54 %), and negative expressions of CD34 (5.78 %) and CD14 (2.46 %). Adipose-derived stem cells treated with conditioned media exhibited adipogenic, osteogenic, and chondrogenic differentiation after staining.

### Clinical and radiological outcomes

The clinical and radiological outcomes are summarized in Table 2. The mean VAS score improved significantly from 7.2 ± 1.0 to 4.7 ± 1.4 in group I and from 7.3 ± 0.8 to 3.7 ± 1.5 in group II at the final follow-up (*P* < 0.001 for both groups). The mean AOFAS score also improved significantly from 61.7 ± 5.8 to 80.9 ± 6.7 in group I and from 60.6 ± 6.1 to 85.2 ± 5.1 in group II at the final follow-up (*P* < 0.001 for both groups). There were significant differences in the mean VAS and AOFAS scores between groups at the final follow-up (*P* = 0.002 and 0.010, respectively).

**Table 2:** Clinical and radiological outcomes in both groups

	Group I	Group II	*P* Value
VAS			
Preoperative	7.2 ± 1.1	7.2 ± 0.8	n.s.
Final follow-up	4.9 ± 1.3	3.7 ± 1.5	.002
AOFAS score			
Preoperative	62.3 ± 6.1	61.0 ± 5.8	n.s.
Final follow-up	81.2 ± 6.2	85.2 ± 5.2	.010
TAS, °			
Preoperative	82.3 ± 2.2	83.0 ± 2.1	n.s.
Final follow-up	89.1 ± 2.1	89.4 ± 1.8	n.s.
TT, °			
Preoperative	5.3 ± 1.1	5.6 ± 1.4	n.s.
Final follow-up	2.4 ± 0.9	2.8 ± 1.1	n.s.
TLS, °			
Preoperative	77.6 ± 1.7	76.9 ± 1.8	n.s.
Final follow-up	79.3 ± 1.4	78.8 ± 1.8	n.s.

Values are expressed as mean ± standard deviation. *VAS* visual analog scale, *AOFAS* American Orthopaedic Foot and Ankle Society ankle-hind foot scale, *TAS* tibial-ankle surface angle, *TT* talar tilt, *TLS* tibial-lateral surface angle

The mean preoperative TAS, TT, and TLS angles were did not differ significantly between groups; all improved significantly at the final follow-up (all *P* < 0.001). However, there were no significant differences between groups at the final follow-up (all n.s.).

### Second-look arthroscopic findings

At the second-look arthroscopy, the following additional arthroscopic procedures were performed: debridement, synovectomy, and adhesiolysis were performed in 58 (29 in group I and 27 in group II), 37 (19 in group I and 18 in group II), and 32 (17 in group I and 16 in group II) ankles, respectively, with no significant differences in distribution.

The ICRS overall repair grades in each group are summarized in Table 3. According to the ICRS overall repair grades, 6.1 % and 38.7 % of lesions in groups I and II, respectively, were grade I or II on the medial aspect of the talar dome. Similarly, regarding the medial aspect of the tibial plafond, 6.1 % and 35.5 % of lesions in groups I and II, respectively, were grade I or II. Regarding the articular surface of the medial malleolus, grade I or II lesions were observed only in group II (11.9 %). The overall ICRS grades were better in group II than in group I (Fig. 2); there were significant differences in ICRS grades between groups regarding the medial aspect of the talar dome (*P* = 0.015), medial aspect of the tibial plafond (*P* = 0.044), and articular surface of the medial malleolus (*P* = 0.005).

**Table 3:** ICRS repair grades at second-look arthroscopy according to the location of cartilage lesion in both groups

	Medial talar dome			Medial tibial plafond			Articular surface of MM		
	Group I	Group II	*P*	Group I	Group II	*P*	Group I	Group II	*P*
Grade			.015			.044			.005
I	0	1 (3.2)		0	1 (3.2)		0	1 (3.2)	
II	2 (6.1)	11 (35.5)		2 (6.1)	10 (32.3)		0	3 (9.7)	
III	18 (54.5)	11 (35.5)		20 (60.6)	12 (38.7)		17 (51.5)	21 (67.7)	
IV	13 (39.4)	8 (25.8)		11 (33.3)	8 (25.8)		16 (48.5)	6 (19.4)	

Values are expressed as number (%). *MM* medial malleolus, *ICRS* International Cartilage Repair Society

### Correlations among clinical and radiological outcomes and ICRS grades

ICRS grades at second-look arthroscopy were significantly correlated with clinical outcomes at the time of second-look arthrosocpy in both groups (all *P* < 0.05) (Table 4). In other words, as the quality of repaired cartilage increased, the VAS and AOFAS scores increased significantly in both groups. However, there were no correlations of postoperative radiological outcomes with clinical outcomes at the final follow-up or ICRS grades at the time of second-look arthroscopy (Table 5).

**Table 4:** Correlations between ICRS repair grades at second-look arthroscopy and clinical outcomes at the time of second-look arthroscopy in both groups

	VAS				AOFAS			
	Group I		Group II		Group I		Group II	
	S rho	*P*	S rho	*P*	S rho	*P*	S rho	*P*
ICRS grade								
Medial talar dome	0.398	.022	0.568	.001	−0.602	<.001	−0.729	<.001
Medial tibial plafond	0.363	.038	0.532	.002	−0.604	<.001	−0.796	<.001
Articular surface of MM	0.395	.023	0.371	.040	−0.550	.004	−0.585	.001

Values expressed were obtained using the Spearman’s rank-order correlation test. *ICRS* International Cartilage Repair Society, *VAS* visual analog scale, *AOFAS* American Orthopaedic Foot and Ankle Society ankle-hind foot scale, *S rho* Spearman rho, *MM* medial malleolus

**Table 5:** Correlations between postoperative radiological outcomes and clinical outcomes at final follow-up and ICRS grades at second-look arthroscopy

	TAS		TT		TLS	
	S rho	*P*	S rho	*P*	S rho	*P*
VAS	0.112	n.s.	−0.201	n.s.	0.276	n.s.
AOFAS score	−0.197	n.s.	0.161	n.s.	−0.237	n.s.
ICRS grade						
Medial talar dome	0.062	n.s.	−0.187	n.s.	0.218	n.s.
Medial tibial plafond	0.174	n.s.	−0.247	n.s.	0.146	n.s.
Articular surface of MM	0.080	n.s.	−0.123	n.s.	0.118	n.s.

Values expressed were obtained using the Spearman’s rank-order correlation test. *ICRS* International Cartilage Repair Society, *TAS* tibial-ankle surface angle, *TT* talar tilt, *TLS* tibial-lateral surface angle, *S rho* Spearman rho, *VAS* visual analog scale, *AOFAS* American Orthopaedic Foot and Ankle Society ankle-hind foot scale, *MM* medial malleolus

## Discussion

The main finding of this study is that the clinical and second-look arthroscopic outcomes in group II were better compared to those in group I. Although the clinical and radiological outcomes were similarly improved in both groups, cartilage regeneration according to ICRS grades, which was significantly correlated with clinical outcomes (Table 4), was significantly better in those who received MSC injection (Table 3). In addition, postoperative radiological outcomes were not significantly correlated with clinical outcomes or ICRS grades (Table 5). Therefore, the better clinical outcomes in those who received MSC injection with marrow stimulation than those who received marrow stimulation alone may be attributable to better cartilage regeneration; this finding supports our hypothesis that additional MSC injection is helpful for cartilage regeneration in patients with varus ankle osteoarthritis who have undergone arthroscopic marrow stimulation along with SMO.

In this study, we assessed the clinical outcomes in both groups and found that VAS and AOFAS scores improved significantly in group II compared with group I at the final follow-up (*P* = 0.002 and 0.010, respectively) (Table 2). In addition, the SEM in differences between the groups was larger than the SEM of each group. Therefore, we considered that the differences in clinical outcomes at final follow-up between the groups, although they were small differences, were truly significant differences.

From the review of literatures, some authors reported the occurrence of partial remodeling of the articular cartilage with cartilage regeneration after high tibial osteotomy (Fujisawa et al. 1979; Koshino and Tsuchiya 1979). Furthermore, marrow stimulation procedures combined with high tibial osteotomy have been attempted in order to improve cartilage lesion remodeling (Matsunaga et al. 2007; Sterett and Steadman 2004). Several authors reported a positive correlation between cartilage regeneration and the clinical outcomes of high tibial osteotomy (Koh et al. 2014; Koshino et al. 2003; Matsui et al. 1979; Parker et al. 2011). Accordingly, we anticipated that marrow stimulation can improve the outcomes of SMO to a similar extent as high tibial osteotomy. Kim et al. (Kim et al. 2014b) found a significant correlation between cartilage regeneration and the clinical outcomes of SMO in patients with varus ankle osteoarthritis and reported that the arthroscopic marrow stimulation procedure improves cartilage regeneration after SMO. Moreover, Koh et al. (Koh et al. 2014) recently compared the clinical and second-look arthroscopic outcomes of patients undergoing high tibial osteotomy for varus osteoarthritic knee with or without MSC injection and reported better cartilage regeneration with good clinical outcomes in patients with MSC injection than patients without MSC injection. Taking these findings into account, we performed MSC injection to augment marrow stimulation in order to improve the outcomes of SMO for varus ankle osteoarthritis.

Marrow stimulation procedures promote cartilage repair by stimulating bone marrow through the subchondral bone and by producing blood clots containing MSCs on the articular surface (Madry et al. 2010). However, several studies demonstrated that the cartilage regenerated by marrow stimulation mainly has a fibrocartilaginous nature, which is biomechanically insufficient compared to native hyaline cartilage (Breinan et al. 2000; Convery et al. 1972; Kaul et al. 2012; Shapiro et al. 1993). As cartilage primarily serves a biomechanical function, tissue engineering strategies must ultimately produce constructs able to exhibit the most essential mechanical properties of native cartilage (Diekman and Guilak 2013). Therefore, we posited that augmenting marrow stimulation would improve cartilage regeneration and accordingly performed supplementary MSC injection in this study.

MSCs have recently been applied as a valuable adjunct to marrow stimulation for cartilage regeneration. McIlwraith et al. (McIlwraith et al. 2011) injected MSCs into microfractured chondral defects in equine models and reported that MSCs enhanced cartilage repair quality with increased aggrecan content and tissue firmness. Fortier et al. (Fortier et al. 2010) compared the results of cartilage repair in equine models treated with MSCs and microfracture with microfracture alone; they also found MSCs could result in the healing of acute full-thickness cartilage defects to a greater extent than that after microfracture alone. Kim et al. (Kim et al. 2014a; Kim et al. 2013) reported that MSC injection with marrow stimulation resulted in superior clinical outcomes in patients with osteochondral lesion of the talus compared to marrow stimulation alone. Although the present study evaluated joints with osteoarthritis, which are characterized by diffuse degeneration of the articular cartilage and accompanied by subchondral bone sclerosis and synovial inflammation (Gharbi et al. 2011) and also differ from joints with localized chondral defects, a similar mechanism associated with attempted repair processes might be mediated at least in part of the microenvironment by MSC injection. We evaluated cartilage lesions after marrow stimulation with SMO by second-look arthroscopy; the results show the ICRS overall repair grades were significantly better in patients who received MSC injection with marrow stimulation than those who received marrow stimulation alone (Table 3). In addition, ICRS grades at second-look arthroscopy were significantly correlated with clinical outcomes at the final follow-up (Table 4). Furthermore, the clinical outcomes of patients who received MSC injection with marrow stimulation were significantly better than those who received marrow stimulation alone (Table 2). These results suggested that MSC injection contributed to improved cartilage regeneration, which resulted in better clinical outcomes.

The present study has some limitations. First, the number of patients was relatively small, the follow-up period was short, and the data were collected retrospectively. Therefore, a large randomized prospective study with a longer follow-up period comparing marrow stimulation with or without MSC injection is required to more accurately evaluate the effect of MSCs. In addition, because the decision whether to receive MSC injection at the time of surgery was solely up to the patients, there might be the problem of selection bias in this study. From this point of view, we compared the baseline demographics between patients who did not receive the second-look arthroscopy and patients who underwent the second-look arthroscopy, and found no significant differences in basic characteristics between the patients groups regarding patient age, sex, body mass index, side of involvement, follow-up period, VAS score for pain, and AOFAS score. Therefore, given that no similar studies of this size have been published, we believe that these data are valuable for comparing the outcomes between MSC injection with marrow stimulation and marrow stimulation alone in patients with varus ankle osteoarthritis who have undergone SMO.

First, the number of patients was relatively small, the follow-up period was short, and the data were collected retrospectively. Therefore, a large randomized prospective study with a longer follow-up period comparing marrow stimulation with or without MSC injection is required to more accurately evaluate the effect of MSCs. In addition, because the decision whether to receive MSC injection at the time of surgery was solely up to the patients, there might be the problem of selection bias in this study. Second, we used VAS and AOFAS scores to evaluate clinical outcomes and ICRS grades to investigate the second-look arthroscopic outcomes after MSC injection with marrow stimulation combined with SMO. It is important to examine the mechanical properties and biological functions of regenerative cartilage and compare them with those of native cartilage. Therefore, further studies involving histologic evaluation in conjunction with clinical and arthroscopic outcomes and power analysis are required to clarify the effect of MSC injection. Third, the second-look arthroscopy was performed 1 year postoperatively. It is unknown how the repaired cartilage will behave over the long term, and changes in the influential factors after the first postoperative year cannot be predicted at present. Furthermore, the ICRS grades at second-look arthroscopic surgery were assessed by the surgeon. Therefore, to avoid potential bias, assessment of the ICRS grade by an independent observer who is blinded to the surgical technique is required for a more objective evaluation. In addition, a power analysis with inter-observer variability in the articular cartilage grading system would be useful in future studies. We added these sentences in the limitation section. Fourth, the loss of correction after SMO could influence the clinical outcome. Although there was no significant correlation of postoperative radiological outcomes with clinical outcomes at the final follow-up and ICRS grade (Table 5), the long-term radiological outcomes might influence the clinical outcomes. Finally, the optimal number of MSCs to be applied remains unknown.

## Conclusions

The clinical and second-look arthroscopic outcomes of MSC injection with marrow stimulation were better compared to those of marrow stimulation alone in patients with varus ankle osteoarthritis who have undergone SMO. Furthermore, the ICRS grade is significantly correlated with clinical outcome.
